# Antioxidant Property of the Egyptian Propolis Extract Versus Aluminum Silicate Intoxication on a Rat’s Lung: Histopathological Studies

**DOI:** 10.3390/molecules25245821

**Published:** 2020-12-10

**Authors:** Ali H. Abu Almaaty, Yasmin M. Abd El-Aziz, Nahed A. Omar, Ahmed M. Abdeen, Hala Afifi, Tarek S. Ibrahim, Sameh S. Elhady, Amgad I. M. Khedr

**Affiliations:** 1 Department of Zoology, Faculty of Science, Port Said University, Port Said 42526, Egypt; ali_zoology_2010@yahoo.com (A.H.A.A.); yasminabdelaziz2012@yahoo.com (Y.M.A.E.-A.); 2 Department of Zoology, Faculty of Science, Damietta University, Damietta 34511, Egypt; nahedomar2000@yahoo.com; 3 Department of Zoology, Faculty of Science, Mansoura University, Mansoura 35516, Egypt; eabdeen1@gmail.com; 4 Pharmacy Department, College of Health Sciences, City University College of Ajman, Ajman 18484, UAE; a.hala@cuca.ae; 5 Department of Pharmaceutical Chemistry, Faculty of Pharmacy, King Abdulaziz University, Jeddah 21589, Saudi Arabia; tmabrahem@kau.edu.sa; 6 Department of Pharmaceutical Organic Chemistry, Faculty of Pharmacy, Zagazig University, Zagazig 44519, Egypt; 7 Department of Natural Products, Faculty of Pharmacy, King Abdulaziz University, Jeddah 21589, Saudi Arabia; ssahmed@kau.edu.sa; 8 Department of Pharmacognosy, Faculty of Pharmacy, Port Said University, Port Said 42526, Egypt; 9 Department of Pharmacognosy, Faculty of Pharmacy, Horus University-Egypt, New Damietta 34518, Egypt

**Keywords:** aluminum silicate, propolis, rat lung, biochemical parameters, histopathological investigations

## Abstract

In this study, we evaluated the inflammatory responses induced by aluminum silicate (AS) cytotoxicity in rat lungs. The prophylactic effect of propolis extract was evaluated in 60 adult male albino rats. The rats were divided into six groups: (1) a normal, healthy control group; (2) a normal group fed with 200 mL of propolis extract/Kg; (3) a low-dose positive control group injected with 5 mg/kg of AS; (4) a treated group given propolis and a low dose of AS; (5) a high-dose positive control group injected with 20 mg/kg of AS; and (6) a treated group given propolis with a high-dose of AS. At the end of the two-month experiment, the rats’ lungs were removed. For each pair of lungs, one portion was subjected to biochemical analysis and the other underwent hematoxylin and eosin (H&E) staining in order to study its histology. The rats that received AS doses displayed significant disorders in their antioxidant contents as well as in their enzymatic activities and their histopathological structures revealed severe damage to their lung tissues. Upon the rats being treated with propolis, the enzymatic and antioxidant contents improved and partial improvements in the lung structures appeared, including minimized congestion, a reduced hemorrhage of blood vessels and preserved bronchioles, alveolar ducts, and alveoli. The prophylactic effectiveness of propolis extract on the cytotoxicity of AS, owing to the antioxidant properties of propolis, were studied.

## 1. Introduction

Today, the third most plentiful element used globally is aluminum (Al), which represents 8% of the total compounds in world ecology. It is the most widely distributed metal in the environment and is highly used in daily life, resulting in frequent exposure to human beings [1,2]. Moreover, alumino-silicates, clays and feldspars are considered to be major components of a large number of minerals that are found in the environmental crust [3]. Generally, aluminum is widely distributed in most plant and animal tissues. Al has variable uses, including in industrial processes, in food processing, in ceramic pot and porcelain dish making and in the processing of products such as stones, glasses, clays and jewels. The prevalence of Al has significantly toxic implications on humans who depend on these products [4,5]. The lung directly receives Al, particularly in the form of motes of aluminum, along with silicate and the particles of other compounds. A significantly higher concentration of Al appears in lung tissues, which concentrates over time in living organisms, more so than in other organ tissues [6]. Aluminum silicate is also found in vaccinations, antacid medications and antiperspirants, from which the element enters the human body [1,4,7,8,9]. Silicon dioxide (silica) also plays an important role in the solubility of alumino-silicate minerals. These minerals, generally, are found as feldspars in volcanic or igneous rock and comprise two thirds of all minerals [7]. Crystalline silica toxicity has been directly connected to these minerals as they have variable carcinogenic, fibrogenic and mutagenic potencies [10]. The gastrointestinal and respiratory tracts of living organisms are impacted by the absorption of aluminum [11]. Many workers who deal with aluminum are prone to respiratory diseases and breathing impairments, which can result in chronic obstructive pulmonary disease [12,13]. Aluminum can impact human organs, tissues and cells, such as the alveolar of pulmonary tissues, renal tissues, hepatocytes, blood, the hippocampus and the cerebrum [14,15,16]. Moreover, chronic or long-term exposure to aluminum silicate leads to the sedimentation of the metal in the lung as well as in other tissues and organs, causing many metabolic disorders, histological damages, membrane changes and alterations to diseases [17,18,19]. Exposure to aluminum can also lead to the production of free radicals in the body. Aluminum exposure also results in the down regulation of lung antioxidant concentrations in non-cancerous pulmonary diseases, such as asthma and other chronic obstructive and inflammatory lung diseases [20].

Propolis comes from the excretions of bees mixed with beeswax and a resinous material collected from various plants during all seasons. Propolis is used for the protection of the beehive against predators and for repairing any openings or tears in the beehive. Previous studies have mentioned that the main components of propolis include more than 300 natural compounds, such as flavonoids, esters, terpenoids, fatty acids, amino acids, polysaccharides, steroids, terpenes, alcohols and hydrocarbons [21]. The different types of flavonoids in propolis may be responsible for its biological and clinical mechanisms [22]. In folk medicine, propolis was known to be used by Greek and Roman physicians as an anti-viral, anti-microbial, anti-ulcer, anti-parasitic, anti-tumor, anti-aging, antioxidant, anti-inflammatory and anti-fatigue treatment, as well as in the regulation of blood lipids and blood sugars, in wound treatment [22,23,24] and as a free radical scavenger [25]. Propolis extract counteracts the cytotoxicity effects induced by aluminum [26]. Medically, propolis extract has been used to regulate anti-oxidative levels without changing blood parameters or raising the pulmonary parameters of asthmatic patients [27].

Unfortunately, there are only a few studies that have described the effects of propolis on the structure of the lung. Furthermore, the use of propolis as a protective and ameliorative supplement against aluminum silicate toxicity needs to be investigated. Therefore, the aim of this study was to investigate the effect of aluminum silicate administration on biochemical oxidative stress, lung enzymatic activity and the histological structure of the lung and to assess any possible protection conferred by the concomitant administration of bee propolis extract.

## 2. Results

### 2.1. Biochemical Oxidative Stress Parameters

**Figure 1:**
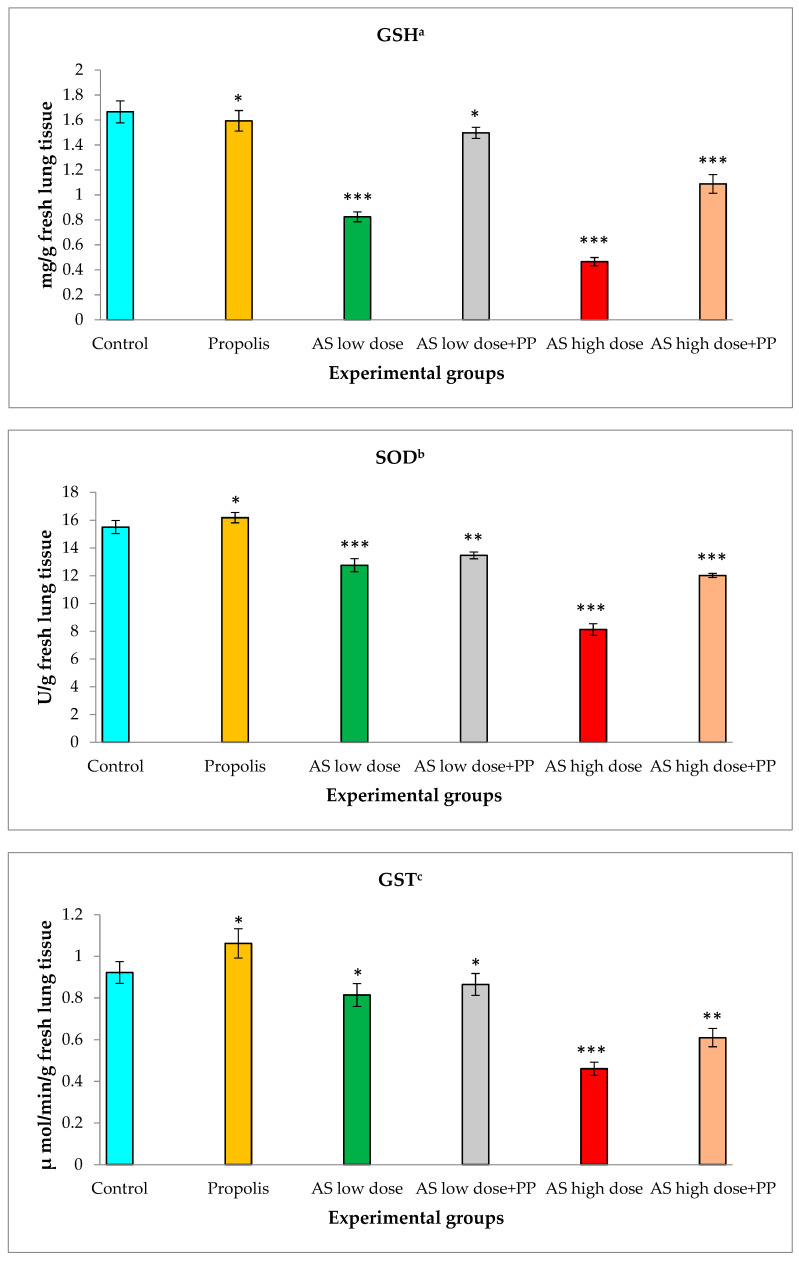
Estimation of biochemical oxidative stress in rat lungs in the different animal groups: (**a**) glutathione reductase (GSH); (**b**) superoxide dismutase (SOD); (**c**) glutathione s-transferase (GST). *—Non-significant, **—Significant, ***—Highly significant.

Antioxidant status was evaluated in lung tissues using glutathione reductase (GSH), superoxide dismutase (SOD) and glutathione s-transferase (GST) (Figure 1). The group of healthy control rats showed normal levels of antioxidant enzymes, similar to the levels achieved with propolis. The levels of biochemical oxidative stress (antioxidant parameters) at a low dose of aluminum silicate revealed significantly lower concentration decreases than in the healthy control group. Briefly, the GSH level was significantly down-regulated from 1.67 ± 0.09 mg/g tissue in the healthy control group to 0.82 ± 0.04 mg/g tissue in the low-dose AS group and to 0.47 ± 0.03 mg/g tissue in the high-dose AS group. The SOD level decreased from 15.51 ± 0.47 U/g in the healthy control group to 12.76 ± 0.47 U/g in the low-dose AS group and to 8.13 ± 0.42 U/g in the high-dose AS group. The measured level of GST decreased from 0.92 ± 0.05 U/g in the healthy control group to 0.81 ± 0.05 U/g in the low-dose AS group and to 0.46 ± 0.03 in the high-dose AS group µmol/min/g in fresh lung tissues.

On the other hand, these effects were modulated by the treatment of propolis (200 mL/kg) on the rat groups with low and high doses of AS. GSH non-enzymatic antioxidant activity rose to 1.50 ± 0.04 mg/g tissue in the low-dose AS group and significantly up-regulated to 1.09 ± 0.08 mg/g tissue in the high-dose AS group. SOD enzymatic antioxidant activity displayed significant increases up to 13.47 ± 0.24 U/g in the low-dose AS group and significant increases up to 12.02 ± 0.15 U/g in the high-dose AS group. GST non-enzymatic antioxidant levels markedly increased to 0.87 ± 0.05 µmol/min/g in the low-dose AS group and significantly up-regulated to 0.61 ± 0.04 µmol/min/g in the high-dose AS group.

### 2.2. Determination of Lung Enzymatic Activities

**Figure 2:**
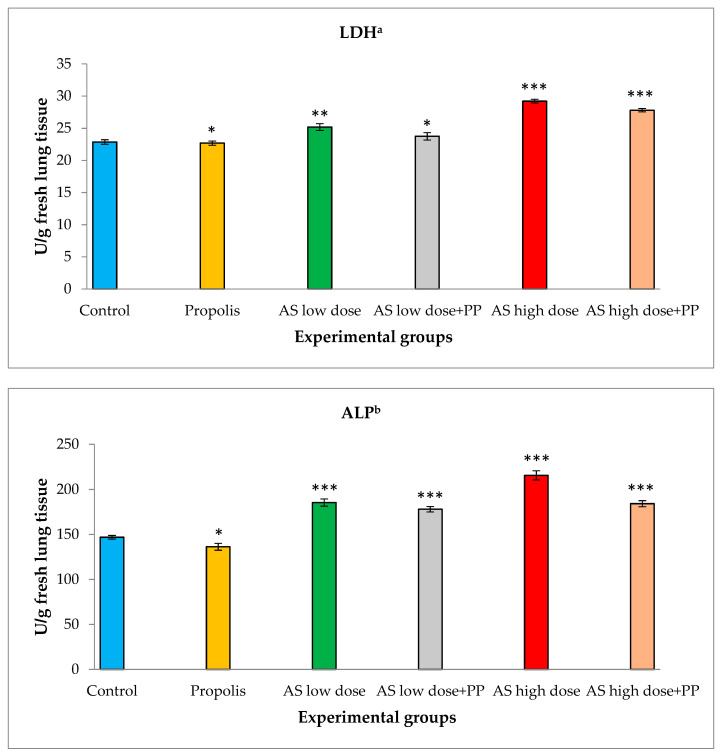
Determination of lung enzymatic activities in rat lungs in the different animal groups: (**a**) for lactate dehydrogenase (LDH); (**b**) for alkaline phosphatase (ALP). *—Non-significant, **—Significant, ***—Highly significant.

The activities of lactate dehydrogenase (LDH) and alkaline phosphatase (ALP) were investigated in the lung tissues of rats (Figure 2). The group of healthy control rats showed normal levels of these enzymes. Similar levels were noted in the presence of propolis extract. The lactate dehydrogenase level was significantly raised in fresh lung tissue from 22.87 ± 0.36 U/g in the healthy control group to 25.20 ± 0.53 U/g in the low-dose AS group and to 29.21 ± 0.30 in the high-dose AS group. These effects were regulated when the low- and high-dose AS groups were treated with propolis extract (200 mL/kg). The LDH activity revealed significant decreases to 23.76 ± 0.58 U/g in the low-dose AS group and significant down regulation to 27.80 ± 0.27 U/g in the high-dose AS group.

Alkaline phosphatase (ALP) levels in fresh lung tissue increased from 146.87 ± 2.02 in the healthy control group to 185.42 ± 3.97 in the low-dose AS group and to 215.70 ± 5.08 U/g in the high-dose AS group. Upon treatment with propolis extract (200 mL/kg), the ALP activity displayed significant decreases to 178.05 ± 2.99 U/g in the low-dose AS group and significant decreases to 184.20 ± 3.36 U/g in the high-dose AS group.

### 2.3. Histological Investigations

**Figure 3:**
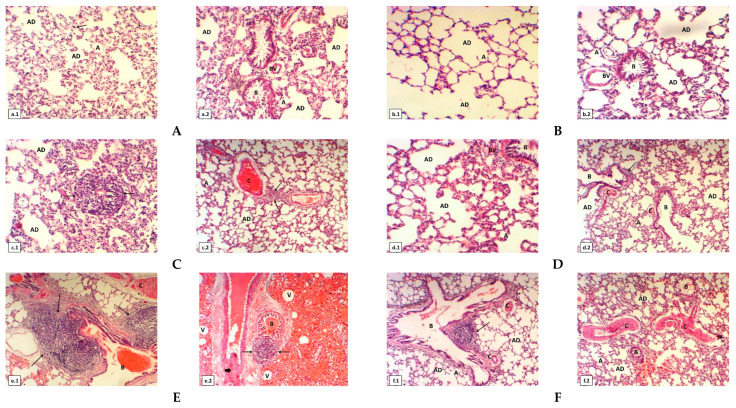
(**A**) Cross section of lung of normal healthy albino rats represents: (**a.1**) normal architecture of lung tissue, alveolar ducts (AD), sacs, alveoli (**A**) and capillary (arrow); (**a.2**) it shows normal bronchioles (**B**), alveolar ducts (AD), sacs and normal blood vessels (BV). (**B**) Cross section of lung of rats which fed on propolis extract: (**b.1**) it shows alveolar ducts (AD) and alveoli (A); (**b.2**) it illustrates normal bronchioles (B) with lumen is empty, alveolar ducts (AD), sacs and normal blood vessels (BV). (**C**) Cross section of another lung of low dose of aluminum silicate (5 mg/kg) induced rats: (**c.1**) it shows numerous lymphocytes (arrows) and alveolar ducts (AD) are noticed; (**c.2**) it shows congestion of blood vessels (C), numerous lymphocytes (arrows), alveolar ducts (AD) and alveoli (A). (**D**) Cross section of another lung of low dose of aluminum silicate (5 mg/kg) and propolis treated rats show clear signs repair of architecture of lung tissue: (**d.1**) it represents the alveolar ducts (AD) and blood vessels (BV) with restoration of normal lung histological architecture; (**d.2**) it shows alveoli (A) with more defined, mild degeneration of the bronchioles (B) and some congestion of blood vessels (C) are founded. (**E**) Cross section of another lungof high dose of aluminum silicate (20 mg/kg) induced rats: (**e.1**) it illustrates a significant accumulation of macrophages and small increases of neutrophils (arrow), formation of eosinophilic hyaline casts inside the lumen of bronchioles (B) and congestion of blood vessels (C); (**e.2**) it displays formation of eosinophilic hyaline casts inside the lumen of bronchioles (B), numerous lymphocytes (arrows) with fibroblastic proliferation replacing lung tissue (thick arrow) and many of vacuoles are visible. (**F**) Cross section of lung of high dose of aluminum silicate (20 mg/kg) and propolis treated rats: (**f.1**) it displays restoration of the bronchioles (B), alveolar ducts (AD), alveoli (A) are mild repair and some congestion of blood vessels (C) are stilled found; (**f.2**) it illustrates clear signs repair of architecture of lung tissue. The bronchioles (B), alveolar ducts (AD), alveoli (A) are mild repair, some congestion and hemorrhage of blood vessels (C) are seen. All cross sections of rat lung tissues stained with H&E (magnification power was 100 X using light microscopy).

The lung tissues of normal healthy control rats (first group) stained with H&E displayed the normal architectural appearance for lung tissue and normal bronchioles, alveolar ducts and sacs, alveoli, capillary and blood vessels, as shown in Figure 3(a.1,a.2). After using the propolis extract as a treatment (second group), the lung tissue structure remained intact, which was observed to be similar to the lung tissue structure of the first group of untreated rats (Figure 3(b.1,b.2)). After rats were injected with a low dose of aluminum silicate (third group), an examination of the lung revealed blood vessel congestion and numerous inflammatory cells (Figure 3(c.1,c.2)). While the group induced with aluminum silicate and treated with propolis extract (fourth group) showed restored lung structure, the diffuse ameliorative appearance of the lung tissue still showed some deterioration and congestion of blood vessels (Figure 3(d.1,d.2)). However, the rats induced with a high dose of aluminum silicate (fifth group) displayed the formation of eosinophilia hyaline casts inside the lumen of the bronchioles, edema, numerous lymphocytes with fibroblastic proliferation replacing lung tissue, the congestion of blood vessels, the accumulation of macrophages, increases of neutrophils, granuloma inflammatory cells and the development of vacuoles (Figure 3(e.1,e.2)). The provision of propolis extract to rats induced with a high dose of aluminum silicate (sixth group) resulted in signs of partial improvement in the appearance of the lung tissue, in which there was minimized congestion and hemorrhage of blood vessels and preserved bronchioles, alveolar ducts and alveoli from the induction of toxicity (Figure 3(f.1,f.2)).

## 3. Discussion

Aluminum silicate (AS) is considered a causative agent of pulmonary cytotoxicity, which alters the alveolar matrix through oxidative stresses that are driven by an imbalance between oxidant and antioxidant defenses [9,28,29,30,31]. In the current study, the inflammatory and the apoptotic effects were observed after using AS at both low and high doses. These effects were measured by testing the antioxidant activity as well as testing the cellular enzymatic production. It was found that the antioxidant activity of GSH, SOD and GST decreased after using AS at a low dose and that the inhibition of the antioxidant activity increased after using a high dose of AS due to the accumulation of free radicals that led to membrane fragility. This finding is in accordance with other authors’ results [9,28,29,30,31]. In our study, treating the models with the natural product, propolis extract, along with low and high doses of AS displayed an ameliorative response of the cellular antioxidant activity. This improvement revealed a decrease of free radicals in the lung cells. The efficacy of propolis extract against acute lung inflammation occurred at a concentration of 200 mg/kg, which revealed its potential effect as an antioxidant and its potential role in inflammatory processes [25]. Propolis extract had a protective effect against bronchial asthma in rat models according to a biochemical study [32]. On the other hand, this study examined LDH and ALP contents in lung tissues, which are expressed during tissue damage. The lung tissues of the tested rats treated with AS showed a marked increase in the release of LDH and ALP. In this study, at a low dose of AS, LDH and ALP enzyme levels increased and were elevated further at a higher dose of AS treatment. When treating these groups of rats with propolis extract, a marked reduction in the level of the enzymes was observed. For more confirmation, LDH levels were found in workers who were frequently exposed to silica dust. The enzyme levels were increased in these workers, which may be due to macrophage damage of the pulmonary lavage [30,33,34]. Interestingly, propolis extract diminished the production of both ALP and LDH.

In the histopathological investigation, it was noticed that the rat lungs treated with a low dose of AS showed some blood vessel hemorrhage and numerous inflammatory granulated and non-granulated cells, such as neutrophils, alveolar macrophages and lymphocytes. At a high-dose of AS, it caused eosinophilia, the formation of hyaline casts inside the cores of lung bronchioles, edema, numerous lymphocytes, neutrophils, inflammatory granular cells, the appearance of fibroblastic spread, the accumulation of macrophages replacing lung tissues and the hemorrhage of blood vessels that pass through the pulmonary alveoli [29,30]. In previous studies, the evaluation of the action of silica on lung tissue was compatible with our results for the cytotoxicity of aluminum silicate [35,36]. From a biological point of view, the treatment of rats with propolis extract after using low and high doses of aluminum silicate was found to be useful for the enhancement of pulmonary tissues, so propolis extract has many ameliorated effects when administered on a regular daily cycle.

Propolis extract can be used as an antioxidant agent against bronchial inflammation in asthmatic rats, it has the potential to inhibit free radicals, it can provide and reactivate antioxidant activities, and it can be used as a treatment to protect from asthma [32]. The effect of propolis in lung tissues as a prophylactic treatment for lung inflammation and damage was reported. The study of propolis extract was in parallel with our findings as propolis extract has prophylactic effects and can ameliorate lung inflammation [37].

## 4. Materials and Methods

### 4.1. The Extract of Propolis

Propolis collection was done from honey hives in Sinai, Egypt. The extraction of propolis was performed according to Cunha [38]. It was administered orally via a stomach gavage tube at a dose of 200 mL/kg.

### 4.2. Experimental Animals

This study was carried out for 60 adult male albino rats (*Rattus norvegicus*) weighing 100–120 g on average. The animal models were obtained from Helwan Animal Station (Ministry of Health, Egypt). All animals were performed according to the Egypt national institute of health guidelines for the care and use of laboratory animals. The study protocol was approved by the animal care and use according to faculty of science committee, Port Said University. The animals were adapted in plastic cages under adequate conditions and allowed water and suitable food (laboratory diet) ad libitum throughout the study in accordance with the international guidelines for the care and use of laboratory animals. They were divided into six groups, with eight rats in each group: the first group was a normal healthy control and did not receive any treatment; the second group was fed 200 mL of propolis extract/kg each day via a stomach gavage tube; the third group was injected with 5 mg/kg of aluminum silicate (Sigma-Aldrich, Saint Louis, MO 63103, USA) (intraperitoneal), considered to be a low dose, twice weekly; the forth group was given propolis extract and aluminum silicate at a low dose, similar to that of the second and third groups; the fifth group was injected with 20 mg/kg of aluminum silicate (intraperitoneal), considered to be a high dose, twice weekly; the sixth group received propolis extract and aluminum silicate at a high dose, similar to that of the second and fifth groups. At the end of two months, the lung organs from each experimented rat were removed for biochemical and histopathological studies.

### 4.3. Biochemical Assay

Rats were euthanized twenty-four hours after the administration of the last dose. The lung organ was isolated then washed with a saline solution (0.9% NaCl). Lung tissues were grinded in ice cold bidistilled water to produce 10% homogenate using a potter-Elvehjen glass homogenizer fitted with a Teflon pestle. The homogenate samples were centrifuged at 5000 rpm for 10 min. The supernatants were aspirated for biochemical investigation. The freshly prepared homogenate samples were used for biochemical assays using a spectrophotometer. The antioxidant biomarkers were evaluated, including superoxide dismutase (SOD) and glutathione reductase (GSH). Antioxidant activities were measured kinetically using a commercially available kit purchased from Bio Diagnostic (Giza, Egypt). According to the manufacturer’s instructions, the assays were done. The results were calculated and expressed as units per milligram of tissue. The content of glutathione S-Transferase (GST) in the lung tissue as an enzyme for detoxification was estimated spectrophotometrically. Moreover, lactate dehydrogenase (LDH) and alkaline phosphatase (ALP) performed as enzymes, indicating inflammation inside the lung cells. GST, LDH and ALP were studied using kits provided by Bio Diagnostic (Giza, Egypt). According to the inserted booklet, estimations were completed.

### 4.4. Histological Study 

Twenty-four hours after the administration of the last dose, the lung portions were washed with a saline solution and then immersed in an aqueous formalin solution (10%) immediately as a fixation step for prevention from lytic enzymes, maceration and other postmortem changes that could degrade the tissue. The lung portions were then dehydrated, cleared and embedded in a paraplast wax block and were cut at 5µ of thickness with a rotating microtome. Samples were stained with H&E and processed for histological examination. The stained lung sections were examined under a light microscope.

## 5. Conclusions

In summary, the prophylactic effect of propolis extracts in 60 adult male albino rats was achieved and the inflammatory responses induced by aluminum silicate (AS) cytotoxicity in rat lungs was also evaluated. Rats that received AS doses displayed significant disorders in their antioxidant contents as well as in their enzymatic activities and their histopathological structures revealed severe damage to lung tissues. Upon treatment with propolis, enzymatic and antioxidant contents improved, lung structures partially improved, the congestion and hemorrhage of blood vessels were minimized and bronchioles, alveolar ducts and alveoli were preserved. The prophylactic effectiveness of propolis extracts against the cytotoxicity of AS is owed to the antioxidant properties of propolis. Further experiments are needed to estimate the clinical uses of propolis extracts and bee products as natural supplements and to determine their curative and prophylactic power in humans.
